# Exercise-Induced Neuroprotection of the Nigrostriatal Dopamine System in Parkinson's Disease

**DOI:** 10.3389/fnagi.2017.00358

**Published:** 2017-11-03

**Authors:** Lijuan Hou, Wei Chen, Xiaoli Liu, Decai Qiao, Fu-Ming Zhou

**Affiliations:** ^1^Exercise Physiology Laboratory, College of Physical Education and Sports, Beijing Normal University, Beijing, China; ^2^Department of Exercise and Rehabilitation, Physical Education College, Hebei Normal University, Shijiazhuang, China; ^3^Department of Pharmacology, University of Tennessee College of Medicine, Memphis, TN, United States

**Keywords:** basal ganglia, dendritic spine, dopamine, glutamate, medium spiny neuron, neuroprotection, neurotrophic factor, physical activity

## Abstract

Epidemiological studies indicate that physical activity and exercise may reduce the risk of developing Parkinson's disease (PD), and clinical observations suggest that physical exercise can reduce the motor symptoms in PD patients. In experimental animals, a profound observation is that exercise of appropriate timing, duration, and intensity can reduce toxin-induced lesion of the nigrostriatal dopamine (DA) system in animal PD models, although negative results have also been reported, potentially due to inappropriate timing and intensity of the exercise regimen. Exercise may also minimize DA denervation-induced medium spiny neuron (MSN) dendritic atrophy and other abnormalities such as enlarged corticostriatal synapse and abnormal MSN excitability and spiking activity. Taken together, epidemiological studies, clinical observations, and animal research indicate that appropriately dosed physical activity and exercise may not only reduce the risk of developing PD in vulnerable populations but also benefit PD patients by potentially protecting the residual DA neurons or directly restoring the dysfunctional cortico-basal ganglia motor control circuit, and these benefits may be mediated by exercise-triggered production of endogenous neuroprotective molecules such as neurotrophic factors. Thus, exercise is a universally available, side effect-free medicine that should be prescribed to vulnerable populations as a preventive measure and to PD patients as a component of treatment. Future research needs to establish standardized exercise protocols that can reliably induce DA neuron protection, enabling the delineation of the underlying cellular and molecular mechanisms that in turn can maximize exercise-induced neuroprotection and neurorestoration in animal PD models and eventually in PD patients.

## Introduction

Parkinson's disease (PD) is a common, age-dependent degenerative neurological disorder caused by a severe loss of the nigrostriatal dopaminergic projection (Kish et al., 1988; Hornykiewicz, 2001; Braak et al., 2004; Kordower et al., 2013), leading to the characteristic motor deficits and symptoms including resting tremor, a slowness and paucity of movements, muscle rigidity, and postural imbalance (Parkinson, 1817; Olanow et al., 2009). Although the dopamine (DA) replacement therapy is effective for relieving the motor deficits, the disease process (Lewy pathology) continues to progress and spread to impair multiple forebrain areas, eventually leading to severe motor function deficits, cognitive impairments, and even dementia (Braak et al., 2004; Katzenschlager et al., 2008; Hawkes et al., 2010; Coelho and Ferreira, 2012; Goedert et al., 2013; Kordower et al., 2013; Del Tredici and Braak, 2016). As the old human population grows due to increased life expectancy, the PD population is also increasing (Zhang et al., 2005; de Lau and Breteler, 2006; Pringsheim et al., 2014), causing an immense suffering to the patients and their families and also creating an enormous economical burden on the society.

There is currently no pharmacological therapy that can modify or slow the disease or protect DA neurons, despite the immense efforts and resources expended in the past five decades since the early 1960s when DA neuron degeneration was first identified as the key pathology of PD (Hornykiewicz, 2001, 2017; Kalia et al., 2015; Bartus and Johnson, 2017a,b). Current pharmacological therapies are capable of only relieving motor symptoms and can not modify or slow the disease progression. Due to the complexity of the disease and using the progress made in the past three decades as a guide, the chances are small in the next three decades for scientists to find a breakthrough pharmacotherapy that can stop, reverse or substantially slow the disease, indicating a major unmet medical need.

**Figure 1:**
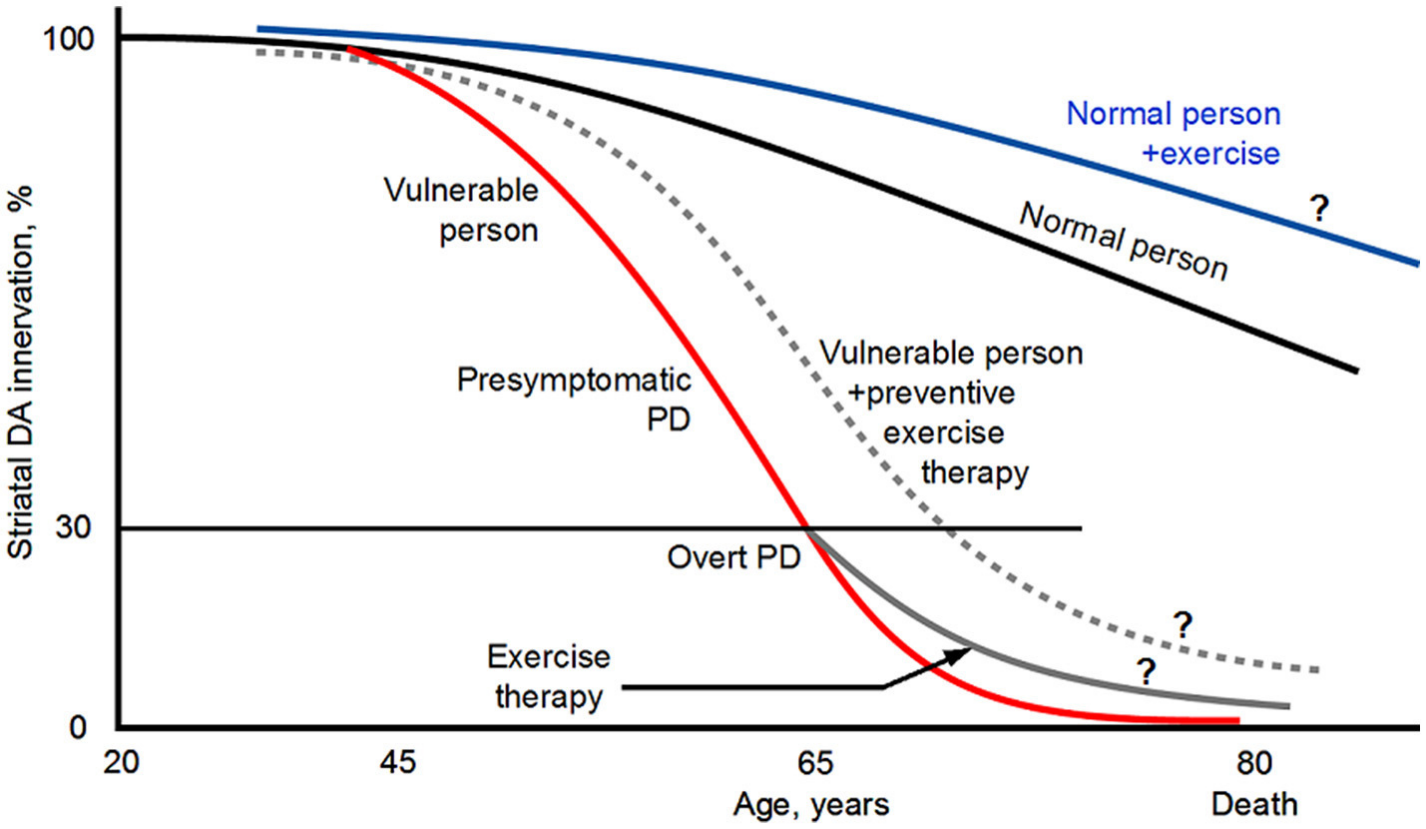
Diagram illustrating that PD has a long presymptomatic period and that exercise may reduce PD risk, delay the appearance of the symptoms, and slow the disease progression. ? indicates that the illustrated possibilities are suggested by indirect evidence but direct evidence is lacking. The diagram is based on Kish et al. (1988), Hornykiewicz (2001), Braak et al. (2004), Gaig and Tolosa (2009), Hawkes et al. (2010), Savica et al. (2010), Coelho and Ferreira (2012), Burke and O'Malley (2013), Goedert et al. (2013), Kordower et al. (2013), Del Tredici and Braak (2016), Noyce et al. (2016) and Salat et al. (2016). The curves and values are approximate because published data are incomplete and variable. Original illustration of Fu-Ming Zhou.

Evidence shows that PD has a 10–20 years or even longer presymptomatic phase (Gaig and Tolosa, 2009; Cheng et al., 2010; Hawkes et al., 2010; Savica et al., 2010; Burke and O'Malley, 2013; Noyce et al., 2016; Salat et al., 2016) (Figure 1). This presents an opportunity to modify or slow the pathological progression from presymptomatic phase to overt PD. Thus, in parallel to the continued search for pharmacotherapies, finding alternative, non-pharmacotherapies is highly necessary to delay and slow the DA neuron degeneration and hence the appearance of the PD symptoms. After the appearance of PD symptoms, non-pharmacotherapies can complement existing and future pharmacotherapies to better control the motor and non-motor (cognitive) symptoms and slow the worsening of the PD symptoms.

Exercise may be a non-pharmacological treatment for presymptomatic and clinical PD that is non-invasive, does not have side effects but has proven benefits for multiple organ-systems (Vina et al., 2012; Petzinger et al., 2013; Burley et al., 2016; Jackson et al., 2016; Lauzé et al., 2016). In this review, we will first briefly summarize the epidemiological and clinical evidence showing the benefits of exercise for PD patients; then we will focus on the basic science aspects of exercise's benefits in PD, particularly the striatum and nigrostriatal DA projection, as they are critically important to movement control. We will summarize, discuss, synthesize clinical, and basic science evidence that exercise may have neuroprotective effects on the nigrostriatal DA system. Equally, important, we will also discuss the critical knowledge gaps in the field where future research is needed to move the field forward and to produce clinically actionable basic science results to benefit PD patients.

## Epidemiological evidence for a potential exercise-induced protection against parkinson's disease

In 1992, Sasco et al. published the results of long term longitudinal epidemiological study on a potential association between the occurrence of PD and physical exercise in 50,002 men who attended Harvard University in Cambridge, Massachusetts, or the University of Pennsylvania in Philadelphia, Pennsylvania, between 1916 and 1950 and were followed up into adulthood for disease and mortality information. This study found that having done regular physical exercise in college was associated with a lower risk for PD, providing the first scientific evidence that exercise may provide a protection against PD (Figure 1).

Since the pioneering study of Sasco et al. (1992), several follow-up large sample epidemiological studies have also examined the correlation between physical activities in early adulthood and PD occurrence in later years. The general conclusion from these epidemiological studies indicate that PD occurrence is lower in people with moderate to vigorous general physical activity and recreational activity (e.g., running, swimming, tennis, bicycling, aerobics, dancing), suggesting that physical activity in early adulthood may reduce PD risks in later years (Chen et al., 2005; Thacker et al., 2008; Xu et al., 2010; Sääksjärvi et al., 2014; Yang et al., 2015; Shih et al., 2016) (Figure 1). Since the exercise and physical activity started a long time (>2–3 decades) before the usually old onset age (~65 years), it appears that the beneficial effects probably starts to protect the DA and basal ganglia (BG) systems long before PD clinical symptoms start and when the DA neuron degeneration is just beginning such that the cellular damages may be more repairable. Certainly, these studies can not exclude the small possibility that people predisposed to PD are physically less active. We also need to note here that one low sample study found no correlation between physical activity and PD risks (Logroscino et al., 2006). Thus, taken together, the majority of the epidemiological data indicate that physical exercise in early adulthood reduces PD risk (Figure 1).

## Exercise benefits for PD patients: clinical evidence

Clinical studies have examined potential therapeutic effects of exercise on PD and reported positive results. For example, 50 min treadmill exercise, three times a week for 3 months improved gait and locomotion speed in PD patients (Shulman et al., 2013). Another study reported that aerobic exercise training improved motor function and motor learning in PD patients (Duchesne et al., 2015). In a large sample study based on National Parkinson Foundation patient database, regular exercise was associated with better quality of life, mobility, and physical function, slower symptom progression, less caregiver burden and less cognitive decline (Oguh et al., 2014; Rafferty et al., 2017). It has also been reported that a 24-week Tai Chi exercise improved balance and gait function while reducing falls in PD patients (Li F. et al., 2012, 2014), although another study indicated that a 16-week Tai Chi exercise failed to produce detectable beneficial effect on the motor function in PD patients (Amano et al., 2013). Additional studies have indicated that that exercise can play an important role in slowing the physical and cognitive decline resulting from PD (Corcos et al., 2013; LaHue et al., 2016; Reynolds et al., 2016; Dipasquale et al., 2017). Physical exercise has also been reported to produce synergistic benefits with L-dopa for improving motor functions in PD patients (Kang et al., 2012).

Taken together, these clinical and epidemiological studies have provided evidence supporting the conclusion that exercise not only has a significant preventive effect on PD, but also has therapeutic value by reducing the symptoms and slowing the symptom and disease progression (Figure 1), and should be promoted to the general population, PD vulnerable population in particular (Burley et al., 2016; Jackson et al., 2016; Lauzé et al., 2016). These studies also indicate that physical exercise needs to be prescribed to PD patients and be an essential component of the treatment for PD (Ahlskog, 2011; Vina et al., 2012; Shulman et al., 2013; Bloem et al., 2015; Pedersen and Saltin, 2015; LaHue et al., 2016; Lauzé et al., 2016; Reynolds et al., 2016) (Figure 1). Indeed, the highly respected International Parkinson and Movement Disorder Society has designated exercise as an adjunct therapy for PD (Fox et al., 2011).

Although much remains to be understood, exercise's benefits for PD are likely to be mediated partly by exercise-induced improvement of the general health (e.g., increasing the cardiovascular and cerebrovascular function), the function of the skeletal musculature and also the function of the broad motor control neural systems including cerebral motor cortices, BG, the cerebellum and the thalamus (Beall et al., 2013; Petzinger et al., 2013; Singh et al., 2014; Wang et al., 2015a,b; Alberts et al., 2016; Burley et al., 2016; Jackson et al., 2016; Shah et al., 2016). Additionally, because of the known importance of the nigrostriatal DA system and the cortico-BG circuitry in motor function, a large number of studies have examined how exercise directly affects these neural systems. Below, we will summarize and synthesize these studies, starting with describing the basic anatomy and physiology of the nigrostriatal DA system and cortico-basal ganglia circuits, likely key neural targets for exercise intervention.

## Anatomy of the nigrostriatal DA system and the basal ganglia

### Components of the basal ganglia and the nigrostriatal DA system

**Figure 2:**
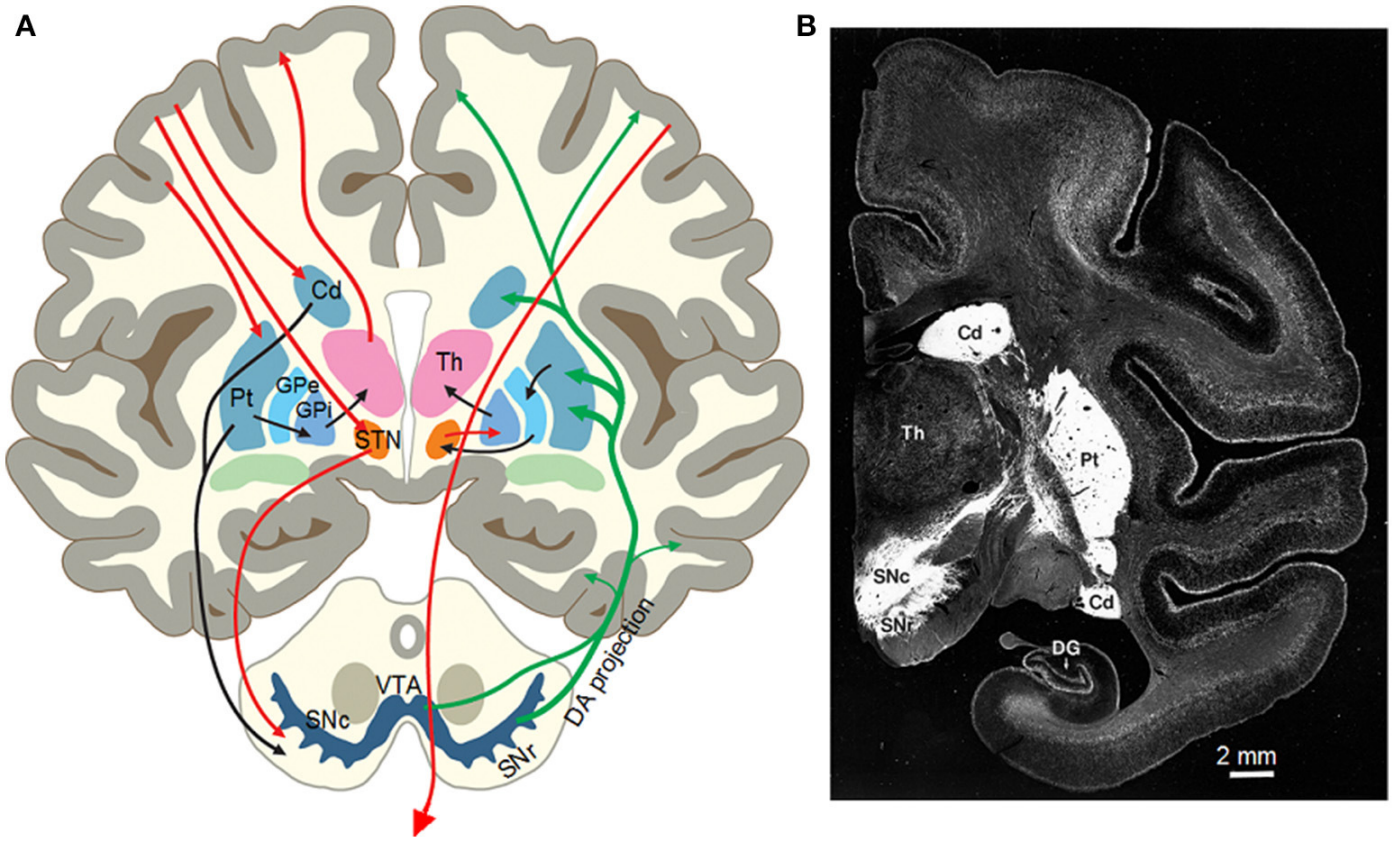
**(A)** Diagram of the nigrostriatal system and the basal ganglia circuitry of the human brain. From Zhou et al. (2003) with permission. **(B)** Photograph of a monkey brain coronal section showing the intense DA innervation in the striatum (the putamen and the caudate nucleus) indicated by DAT immunostain. Modified from Lewis et al. (2001) with permission.

The basal ganglia (BG) are a group of interconnected subcortical nuclei including the striatum, globus pallidus external segment (GPe) and internal segment (GPi), the subthalamic nucleus (STN), the substantia nigra pars compacta (SNc), and pars reticulata (SNr) (Gerfen and Bolam, 2017; Zhou, 2017) (Figures 2A,B). In primates, the striatum comprises the caudate nucleus and putamen. The striatum is the major input nucleus for the BG receiving glutamatergic inputs from motor and somatosensory cortices and other cortical areas (Deng et al., 2015) and the thalamus (Smith et al., 2014). The GABAergic medium spiny neurons (MSNs), comprising 90% of the striatal neurons, are the projection neurons of the striatum and critical to multiple important brain functions as indicated by the profound behavioral consequences of Huntington's disease in which MSNs are lost (Glass et al., 2000) and PD in which striatal DA innervation is lost (Kish et al., 1988; Kordower et al., 2013). One group of MSNs heavily express DA D1 receptors (D1Rs) and project to and inhibit the high frequency firing GABAergic neurons in the GPi and the SNr, the output nuclei of the basal ganglia, forming the direct pathway (Gerfen and Bolam, 2017; Zhou, 2017). The other group of MSNs heavily express D2Rs and project to and inhibit the high frequency firing GABAergic neurons in the GPe, forming the indirect pathway. The high frequency firing GPe GABAergic neurons project to and inhibit the glutamatergic STN neurons (Figure 2A). The STN comprises of a cluster of spontaneously firing glutamatergic neurons that excite GPi/SNr GABAergic neurons (Parent and Hazrati, 1995; Nambu, 2008, 2011; Kita and Kita, 2011; Sano et al., 2013).

A distinct feature of the basal ganglia is the nigrostriatal DA projection and the intense DA innervation in the striatum (Figure 2B). Though the number of cell somata is quite small (Oorschot, 1996; Hardman et al., 2002), the projection axons of the midbrain DA neurons bifurcate repeatedly in the striatum, eventually forming an extremely dense DA axon network in rodents and primates including humans (Levey et al., 1993; Ciliax et al., 1999; Prensa et al., 2000; Lewis et al., 2001; Matsuda et al., 2009; Ding et al., 2015; Morigaki and Goto, 2016). Similarly, the expression levels of D1Rs and D2Rs in the striatum are also extremely high, the highest in the brain in both rodents and primates including humans (Levey et al., 1993; Yung et al., 1995; Hurd et al., 2001; Zhou, 2017), providing the anatomical and molecular substrates for intense DA signaling in the striatum and for DA's profound behavioral effects including motor stimulation.

### Motor function of the striatum and the nigrostriatal DA system

The striatum and the segregated D1-MSN and D2-MSN pathways together with the intense DA regulating system process and integrate sensory, motor, cognitive, and motivational information, and then produce output signals to feedback to cortical, subcortical, and brainstem areas (Tremblay et al., 2015; Haber, 2016). Thus the striatum can affect these motor and non-motor behaviors, and loss of the DA regulation may render the striatum and hence motor and cognitive control mechanisms dysfunctional. The motor function of the striatum and the MSNs is clearly demonstrated when the striatum becomes dysfunctional and loses its normal function, e.g., the abnormal choreic movements in Huntington's disease (Walker, 2007), likely due to the loss of MSNs, especially those in the indirect pathway (Mitchell et al., 1999; Glass et al., 2000; Walker, 2007; Obeso et al., 2014). Consistent with these clinicopathological data, experimental ablation or inactivation of indirect pathway MSNs increases motor activity (Sano et al., 2003, 2013; Durieux et al., 2009, 2012; Bateup et al., 2010; Chiken et al., 2015). It is now clear that D2-MSN activity and the striatopallidal output inhibit movement (hence movement disinhibition in PD), and D1-MSN activity and the consequent striatonigral output facilitate movement (Kravitz et al., 2010; Cui et al., 2013; Sano et al., 2013; Friend and Kravitz, 2014; Jin et al., 2014).

DA activity in the striatum is absolutely required for normal motor function in both animals and humans. This is demonstrated by the fact that local drug infusion into the striatum to block striatal DA receptors induce PD-like akinesia (Franco and Turner, 2012). Local toxin infusion lesioning the striatal DA innervation also leads to motor deficits in animals (Lee et al., 1996; Kirik et al., 1998; Bagga et al., 2015; Willard et al., 2015). In humans, DA neuron degeneration leads to PD (Hornykiewicz, 2001, 2017). The accidental destruction of the nigrostriatal DA system in 1-methyl-4-phenyl-1,2,3,6-tetrahydropyridine (MPTP)-poisoned patients and their motor deficits have provided further confirmation (Ballard et al., 1985; Vingerhoets et al., 1994). These results are consistent with the fact that DA innervation and DA receptor expression are highly concentrated in the striatum (Levey et al., 1993; Yung et al., 1995; Gerfen and Bolam, 2017; Zhou, 2017) (Figures 2A,B). Thus, the DA system's main motor-promoting function is mediated by D1-MSNs and D2-MSNs, although dopaminergic activity in other brain areas may contribute additional complexity to motor control and parkinsonism. These conclusions are further supported by the facts that a total inhibition of L-dopa synthesis in brain DA neurons leads to akinesia and being to unable to feed and drink (Zhou and Palmiter, 1995). Inhibition of DA release by blocking action potential propagation of the nigrostriatal DA axons in the medial forebrain bundle also induces akinesia (Galati et al., 2009).

## Exercise effects on the nigrostriatal DA system in animal PD models

Many studies have investigated the potential beneficial effects of exercise on the nigrostriatal DA system and motor function in animal models of PD, usually toxin-induced DA denervation rodent models. Large amounts of positive results have been obtained, but negative results have also been reported. The main findings from these studies are listed in Table 1 and summarized and discussed below.

**Table 1:** Exercise effects on the nigrostriatal DA system in animal models of PD.

**Study**	**Animal model**	**Exercise type**	**Exercise start timing**	**Treadmill speed or other parameters**	**Exercise duration min/day**	**Total days of exercise**	**Effect on nigral DA neurons**	**Effect on striatal DA axons**	**Effect on striatal DA content**	**Effect on striatal TH content**
**POSITIVE RESULTS**										
Tillerson et al., 2001	Rat 6OHDA	Forced limb use	24 h after 6-OHDA lesion	Not applicable		Up to 28 days	Reduces DA neuron loss	Reduces DA axon loss	Up	Up
Tillerson et al., 2002	Rat 6OHDA	Forced limb use	24 h after 6-OHDA lesion	Not applicable		Up to 28 days	Reduces DA neuron loss	Reduces DA axon loss	Up	Up
Cohen et al., 2003	Rat 6OHDA	Forced limb use	24 h after 6-OHDA lesion	Not applicable		Up to 28 days	Reduces DA neuron loss	Reduces DA axon loss	Up	Up
Tillerson et al., 2003	Rat 6OHDA	Treadmill	24 h after 6-OHDA lesion	15 m/min	30 min/day	Up to 28 days	Reduces DA neuron loss	Reduces DA axon loss	Up	Up
Yoon et al., 2007	Rat 6-OHDA	Treadmill	1 day after 6-OHDA lesion	At a speed of 2 m/min for the first 5 min, and then 3 m/min for the last 25 min	30 min/days	14 consecutive days	DA neurons↑	DA axons↑	Up	
Gerecke et al., 2010	Mouse MPTP	Running wheel	3 month prior to MPTP administration		4.8 km/days	90 days	Reduces DA neuron loss		Up	Up
Lau et al., 2011	Mouse MPTP	Treadmill	For 1 week before, 5 weeks during, and 12 weeks after the completion of chronic MPTP treatment	5 min at 6 m/min, 5 min at 9 m/min, 20 min at 12 m/min, 5 min at 15 m/min, and 5 min at 12 m/min	40 min/day, 5 days/week	18 weeks	Reduces DA neuron loss		Up	Up
Tajiri et al., 2010	Rat 6OHDA	Treadmill	24 h after the 6-OHDA lesion	11 m/min	5 days/week, 30 min/day	4 weeks (20 days)	Reduces DA neuron loss			
Tuon et al., 2012	Rat 6OHDA	Treadmill	8 weeks pre-6-OHDA lesion	13–17 m/min	3 or 4 days/week, 50 min/48h	8 weeks	Not determined	ND	ND	Up
Sung et al., 2012	Mouse MPTP	Treadmill	1 day after last MPTP lesion	12 m /min	30 min/day, 5 days/week	4 weeks	Up	Up		
Real et al., 2013	Rat 6OHDA	Treadmill	1 month, before 6-OHDA		3 days/week		DA neurons↑			
Goes et al., 2014	Mouse 6OHDA	Swimming exercise	4 days after 6-OHDA	2% body weight were attached to the tails	5 times /week	4 weeks			Up	
Smeyne et al., 2015	Mouse MPTP	Running wheel	3 months pre MPTP lesion			3 months	Reduces DA neuron loss		Up	
Tsou et al., 2015	Rat MPP^+^	Treadmill	4 weeks prior to 1-methyl-4- phenylpyridine lesion	12–15 m/min	60 min/day, 5 days/week	4 weeks	Reduces DA neuron loss		Up	
Aguiar et al., 2016a	Mouse 6-OHDA	Treadmill	48 h prior to 6-OHDA lesion	At 16 m/min and speed increased 2 m/min every 3 min until mouse exhaustion	5 times/week	6 weeks	Reduces DA neuron loss	DA axons↑		
Jang et al., 2017	Mouse MPTP	Treadmill	After MPTP lesion	10 m/min	60 min/day, 5 days/week	8 weeks	Up	Up		Up
Koo et al., 2017b	Mouse MPTP	Treadmill	After MPTP lesion	10 m/mine	60 min/day, 5 days/week	8 weeks	Up	Up		Up
Koo et al., 2017a	Mouse MPTP	Treadmill	After MPTP lesion	10 m/min	60 min/day, 5 days/week	8 weeks	Up	Up		Up
Garcia et al., 2017	Rat 6OHDA	Treadmill	1 month, before 6-OHDA	10 m/min, 40 min	3 days/week	1 month	Up	Up	Up	
Real et al., 2017	Rat 6OHDA	Treadmill	1 month, before 6-OHDA	10 m/min, 40 min	3 days/week	1 month	Up	Up	Up	
Shi et al., 2017	Rat 6OHDA	Treadmill	24 h post 6-OHDA lesion	11 m/min	30 min/day/, 5 days/week, 4 weeks	4 weeks (20 days)	Reduces DA neuron loss	Reduces DA axon loss		Up
**NEGATIVE RESULTS**										
O'Dell et al., 2007	Rat 6OHDA	Voluntary +forced wheel running	2.5 weeks before 6-OHDA lesion and continued for up to 4 weeks post-lesion (initiated 1 day after lesion	Forced running: 10.5 m/min	2 × 30 min/day	35 days	No effect			
Petzinger et al., 2007	Mouse MPTP	Treadmill	Started 5 days after MPTP lesion	9.2 ± 1.1 m/min during the first week that further increased to 20.5 ± 0.7 m/min in the last week	5 days/week	28 days	No effect		No effect	No effect
Gorton et al., 2010	Mouse MPTP	Treadmill (inclined 5)+ running wheels	Started 5 days after MPTP lesion	6.7 m/min for 30 min-8.5 m/min for 60 min (Treadmill)	30–60 min/day (Treadmill)	30 days			No effect	
VanLeeuwen et al., 2010	Mouse MPTP	Treadmill	Started 5 days after MPTP lesion	9.2 ± 1.1 m/min- 20.5 ± 0.7 m/min	From 30 min/day 2 sessions of 30 min/day; 5 days/week	28 days			No effect	
Kintz et al., 2013	Mouse MPTP	Treadmill	Started 5 days after MPTP lesion	10.0–24.0 m/min	2 × 30 min/day, 5 days/week	28 days			No effect	
Aguiar et al., 2014	Mouse MPTP	Running wheels	Started 6 weeks prior to MPTP lesion			6 weeks			No effect	
Toy et al., 2014	Mouse MPTP	Treadmill	Started 5 days after MPTP lesion	10.0–24.0 m/min	2 × 30 min/day, 5 days/week	6 weeks			No effect	
Sconce et al., 2015	Mouse MPTP	Running wheels	2 weeks after the last dose of MPTP	Not determined		4 weeks		No effect		
Aguiar et al., 2016b	Mouse MPTP	Treadmill	6 week plus 48 h prior to MPTP administration	11.4 m/min for 25, 30, and 45 min during first three weeks and 13.5 m/min for 25, 30, and 45 min during last three weeks	5 times/week	6 weeks	No effect	No effect	No effect	
Hood et al., 2016	Mouse MPTP	Treadmill	3 weeks after the last dose of MPTP	10.8 m/min	60 min/day, 5 days/week	4 weeks	No change	No effect		No effect
Churchill et al., 2017	Mouse MPTP	Treadmill	4-week progressive MPTP, after last dose of MPTP	18 cm/s	60 min/day, 5 days/week	4 weeks	No significant recovery	No significant recovery		No significant recovery

### Positive results on exercise's neuroprotective effects on the nigrostriatal system

Since the key pathology causing the motor deficits in PD patients and PD animal models is a severe loss of the nigrostriatal DA projection (Kish et al., 1988; Hornykiewicz, 2001; Franco and Turner, 2012; Li et al., 2015), an obvious important question is if the exercise-induced motor benefits in PD animal models and patients is at least partially mediated by a preservation or protection of residual nigrostriatal DA neurons, in addition to exercise's beneficial effects directly on skeletal musculature. So far, there is no study that has directly investigated this question in PD patients for the obvious difficulty in obtaining postmortem tissues in appropriate human subjects. However, many studies have been performed in animal PD models to investigate this question, starting with the publication of the study on the neuroprotective effect of forced limb use on DA neurons in a unilateral 6-OHDA rat PD model in 2001 (Tillerson et al., 2001). So the field is still young.

In their pioneering studies, Schallert and his collaborators exploited the established use-promoting-recovery principle well-known in the neurorehabilitation literature (Jones and Schallert, 1994; Schallert et al., 1997, 2000; Takamatsu et al., 2010; Korol et al., 2013; Mang et al., 2013; Tamakoshi et al., 2014) and made seminal observations about the neuroprotective effects of “forced use” of the DA-lesion impaired forelimb (the limb contralateral to the DA lesion site). “Forced use,” achieved by casting the unimpaired limb 7 days before or immediately after the unilateral MFB 6-OHDA lesion, was needed because the rat does not understand human instruction. They found that forced use of the DA lesion-impaired limb drastically reduced the motor deficits of the impaired limb and also substantially reduced striatal tissue DA content loss (determined by HPLC) (Tillerson et al., 2001, 2003; Cohen et al., 2003). In contrast, *forced non-use* of the impaired limb immediately following the lesion worsened the motor deficits and striatal DA content loss (Tillerson et al., 2002). These results provide evidence that activity of the impaired limb promotes the recovery of the motor deficits and also the recovery of the nigrostriatal DA innervation, or protects DA neurons from 6-OHDA lesion, or both increasing neuroprotection and recovery. These studies laid a foundation for the research field.

Details of their experimental protocol are important. In their rat model with the casting/forced use protection, 6-OHDA lesion reduced the striatal DA content to ~25% of the normal level (Tillerson et al., 2001). This indicates that the lesioned striatum had significant numbers of residual DA axons; these residual DA axons may sprout upon appropriate stimulation and nourishing such as by neurotrophic factors, contributing to the observed DA recovery in the striatum. Such a non-complete DA denervation in the striatum may be necessary to produce a significant neuroprotection and recovery. If the lesion is a total or near DA denervation in a striatal subregion, usually the dorsal striatum, such as in late stage PD, it may be impossible for neuroprotection and recovery to be realized for the DA axon terminals in the dorsal striatum and associated DA neurons.

Additionally, it was shown that these behavior- and DA neuron-protecting effects were reduced when the forced use of the impaired limb was initiated 3 or 7 days after the lesion (Tillerson et al., 2001) (Table 1), indicating an effect of neuroprotection rather than enhancing recovery. Timing of neuroprotective procedures (e.g., exercise) is important. The reduced amphetamine-induced asymmetric rotation in exercised PD animals is another indication of reduced DA denervation and hence reduced DA supersensitivity in the lesioned side (Ungerstedt, 1971a; Schwarting and Huston, 1996). These results provide strong evidence that an appropriately designed exercise treatment regimen can reduce experimental toxin-induced DA neuron lesion and loss that in turn can reduce motor deficits, although exercise may also improve the motor function by improving the general health condition of the animal. These results also suggest that for exercise to have the maximal beneficial effects, it should start early, before the disease (any disease) is diagnosed, particularly when PD is diagnosed, the striatal DA loss has reached 70% and many good opportunities may have been lost. Thus, exercise should start at a young age. These studies laid a strong foundation for studying exercise's neuroprotective effects. Additionally, the forced use of the impaired limb is an excellent experimental design because the impaired limb was forced to performed natural motor activities that the limb was performing before the lesion; perhaps, more importantly, the reported behavioral improvements and DA neuron protection were robust. Although natural forelimb use is difficult to quantify, the practical importance is not diminished because natural limb use can be more easily implemented in experimental animals and humans, and has a high translational value.

The studies discussed above (Tillerson et al., 2001, 2003; Cohen et al., 2003), however, did not examine anatomical improvements of residual and potentially new DA axons in the striatum during the forced use-induced DA neuroprotection and neurorestoration; residual DA neurons and potentially repaired DA neurons (i.e., ghost DA neurons without TH) were also not examined. Future studies are needed to fill these critical knowledge gaps.

Schallert and his collaborators also expanded their forced limb use paradigm in unilateral 6-OHDA lesioned rats to the more quantifiable exercise on treadmill and running wheels in unilateral 6-OHDA rats and bilateral MPTP-lesioned mice, and replicated their earlier results of limb use's (i.e., exercise's) protection of the DA neurons or exercise's promotion of DA neuron recovery from MPTP lesion in mice and 6-OHDA lesion in rats (Tillerson et al., 2003). Since then, other laboratories have performed follow-up studies and confirmed and expanded the original findings on exercise's neuroprotective effects on DA neurons in mouse MPTP and rat 6-OHDA PD models (Tillerson et al., 2003; Garcia et al., 2017; Real et al., 2017; Shi et al., 2017). For example, using intrastriatal 6-OHDA DA lesion in rats, Yoon et al. (2007) and Tajiri et al. (2010) reported similarly strong DA neuron protection in unilateral 6-OHDA rat PD model produced by treadmill exercise that was initiated 1 day before lesion and resumed 24 h after lesion and continued for 2–4 weeks. Exercise-induced neuroprotection of DA neurons has also been reported in MPTP mouse model of PD by Richard Smeyne's group (Gerecke et al., 2010; Smeyne et al., 2015). These authors found that in a pre-lesion unrestricted wheel running exercise regimen Gerecke et al. (2010), 1 month running did not provide any protection, 2 months running provided partial protection, and 3 months running provided complete protection such that nigral DA neuron numbers and striatal tissue DA level became normal, indicating the importance of exercise duration for exercise to induce neuroprotection for DA neurons. Furthermore, Gerecke et al. (2010) also reported that the intensity of exercise is critical for the induction of neuroprotection: In the 3 months pre-lesion wheel running exercise regimen, the full daily dose 7.5 km (18,000 daily wheel revolutions) provided complete neuroprotection for DA neurons against MPTP, 2/3 of the full daily exercise dose (12,000 wheel revolutions or 4.8 km) provided partial neuroprotection, whereas 1/3 of the full daily exercise dose (6,000 wheel revolutions or 2.4 km). These data indicate the importance of exercise intensity in inducing neuroprotection.

**Figure 3:**
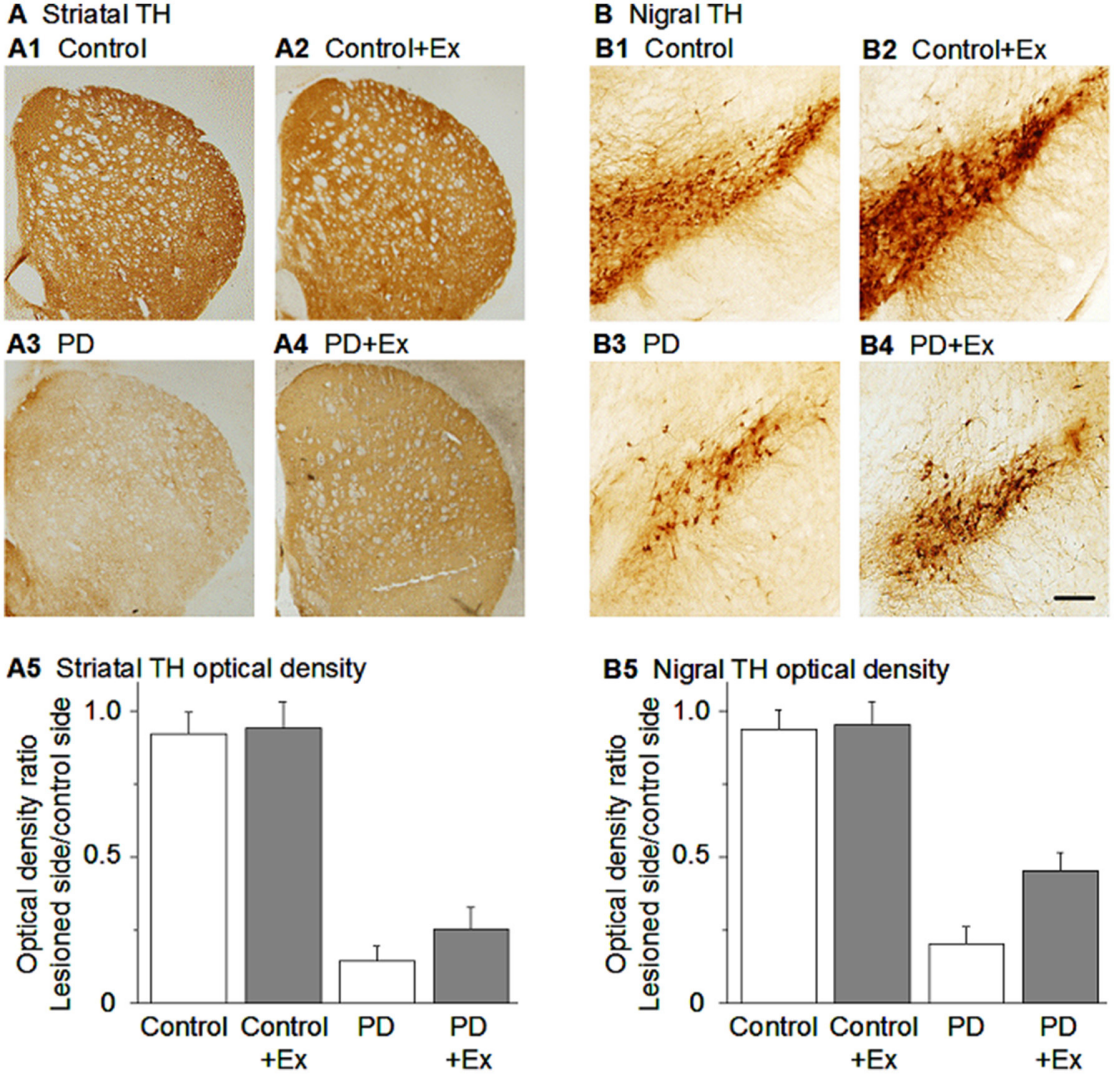
Exercise-induced neuroprotection of DA neurons in the SNc and DA axon terminals in the striatum. **(A)** TH immunostain showing DA axonal innervation under the four conditions. **(B)** TH immunostain showing DA neurons in the substantia nigra under the four conditions. Scale bar in **(B4)**: 0.1 mm for **(A)** and 0.05 mm for **(B)**. Modified from Shi et al. (2017) with permission.

In summary, as listed in Table 1, many studies have reported that in parallel to improved motor function, exercise induced a substantial protection of DA neurons in rodent MPTP or 6-OHDA PD models (Figure 3) (Tillerson et al., 2003; Yoon et al., 2007; Zigmond et al., 2009; Gerecke et al., 2010; Tajiri et al., 2010; Lau et al., 2011; Smeyne et al., 2015; Shi et al., 2017). Additionally, in the rat MPP+ PD model, a 4-week pre-lesion treadmill exercise regime reduced intrastriatal MPP+-induced nigrostriatal DA loss (Tsou et al., 2015).

#### Sources of the new or recovered DA axons

A critical question is where the new or recover DA axons come from. There are four possible sources. The first is the existing, lesioned DA axons. These lesioned DA axons and DA neurons may have suppressed their TH expression such that they become invisible ghost axons and cells in immunostaining examination, and certainly DA level is also reduced, and these lesioned but not destroyed DA cells and axons will recover over time, especially when the animal and hence the DA neurons are young. This recovery of ghost DA neurons and axons has been documented in 6-OHDA and MPTP-lesioned animals (Sanchez-Ramos et al., 1988; Tatton et al., 1990; Lu and Hagg, 1997; Hagg, 1998). Thus, the lesioned DA neurons and axons may transiently lose TH expression and become invisible to TH stain, but these invisible or ghost DA axons and DA cells may re-appear upon appropriate exercise intervention. Future studies need to determine this possibility. The second potential source is the sprouting of the residual DA axons in the striatum. Studies have documented striatal DA axon sprouting after DA toxin lesion in rodents and monkeys (Bezard et al., 2000; Elsworth et al., 2000; Song and Haber, 2000; Stanic et al., 2003a,b). Exercise intervention may promote the sprouting of residual DA axons, contributing to the apparent neuroprotection and recovery. To our knowledge, none of the exercise studies discussed earlier examined anatomical evidence for axonal sprouting; future studies are needed to fill this critical knowledge gap. The third potential source is the generation of local striatal DA neurons. Studies have reported that the striatum can produce local striatal TH-positive, potentially DA-secreting neurons, converted from GABA interneurons after DA denervation, as a compensatory response (Ibáñez-Sandoval et al., 2010), although recent studies indicate that these TH-positive neurons are limited in numbers, do not release DA, and are not functionally significant (Xenias et al., 2015). The fourth potential source is new DA neurons born in the SNc. However, there is currently no convincing evidence that the nigral area can produce new DA neurons in adulthood. Even if new DA neurons are born there, it is not likely that their axons can reach the striatum and repeatedly bifurcate there. So this source is unlikely.

The potential molecular mechanisms underlying exercise-induced neuroprotection and neurorestoration of the nigrostriatal DA system are discussed below in section Molecular Mechanisms Underlying Exercise-Induced Neuroprotection of the Da Neurons and Corticostriatal Circuit.

### Negative results on exercise's neuroprotective effects on the nigrostriatal system

As listed in Table 1, several studies have reported that although improving motor function, exercise intervention did not alter the striatal tissue DA or TH level, nor the numbers of nigral DA neurons and DA axon terminals in the striatum in mouse and rat DA lesion PD models (O'Dell et al., 2007; Petzinger et al., 2007; Gorton et al., 2010; VanLeeuwen et al., 2010; Kintz et al., 2013; Toy et al., 2014; Sconce et al., 2015; Hood et al., 2016; Churchill et al., 2017). For example, using the subactue MPTP C57BL/6J mouse model and a treadmill exercise regime that started 5 days after lesion and lasted for 28 days, Petzinger et al. (2007) and Gorton et al. (2010) reported that exercise did not alter the striatal tissue DA level, although the animals' balance function was improved. In a chronic, progressive MPTP young 2-month adult and also 16-month old mouse model, it has been reported that voluntary wheel running or treadmill exercise did not protect dopamine neurons in the substantia nigra and DA axon terminals in the striatum, but did improve the mouse's paw grip and gait (Sconce et al., 2015; Hood et al., 2016; Churchill et al., 2017). It has also been reported that although not altering tissue DA content, exercise may reduce DAT expression and DA reuptake and thus enhance the residual DA signal, as indicated by the prolonged DA signal monitored by fast cyclic voltammetry, providing another route for exercise to enhance the lesioned, residual nigrostriatal DA system (Petzinger et al., 2007).

These studies reporting a lack of DA neuron protection, however, used relatively low intensity treadmill exercise that was started following a 5-day waiting period after MPTP injection was completed or used voluntary wheel running. As reported in other studies, exercise-induced neuroprotection of DA neurons is critically dependent on the timing of the exercise regimen (Tillerson et al., 2001) and the duration and intensity of the exercise regimen (Gerecke et al., 2010). The use of the 5-day waiting period has probably two reasons: first, the mice are acutely systemically intoxicated in the 5 days following intraperitoneal (IP) injection of MPTP such that exercise is not practical for these mice; second, animal use regulations often impose a 5-day waiting period for MPTP-treated mice. Thus, post-lesion exercise may not be suitable for studying exercise-induced neuroprotection in the mouse MPTP model. Pre-lesion exercise may be a better alternative for mouse MPTP model, as used by Gerecke et al. (2010) and Smeyne et al. (2015). Certainly, the post-lesion exercise with the 5-day waiting period in MPTP mouse model may be used to study the potential neurorestorative effects in the DA and non-DA systems; and beneficial effects may be obtained via non-dopaminergic mechanisms, even if the DA neurons are not protected or restored (Petzinger et al., 2007; Sconce et al., 2015; Hood et al., 2016; Churchill et al., 2017).

### Potential causes of the discrepancy

The studies discussed above indicate a major discrepancy: a large number of studies reported neuroprotective effects of exercise on the DA system, but several studies reported a lack of such a neuroprotective effect (Table 1). At this moment, we do not know the cause(s) of this discrepancy, but the following factors can contribute.

#### DA lesion severity

The more complete the DA axon denervation/destruction, the less likely a neuroprotection, restoration can be obtained. This is because at least a major source of the recovered DA axons/DA neurons are likely from ghost DA axons/neurons and/or DA axon sprouting from residual DA axons (Elsworth et al., 2000). Clinical trials of neuroprotection therapies in PD patients also suggest that neuroprotection/neurorestoration is difficult or impossible to realize when the pathology is too severe or the neurons are too sick or dead to be repaired (Bartus and Johnson, 2017a). However, DA lesion severity data were often not explicitely described such that a determination can not be made.

#### Exercise initiation timing

When initiated before the DA neuron and axons are completely destroyed, exercise may help DA neuron/axons survive and eventually recover. When exercise starts after the DA neuron and axons are completely destroyed, it is too late and no neuroprotection or repair is possible. This idea was supported by the results of Tillerson et al. (2001) that forced use of the lesioned forelimb initiated 24 h after the lesion effectively protected/recovered the striatal DA system, and delayed start of forced use decreased this neuroprotection, a 7-day delay rendered the procedure entirely ineffective. This possibility may contribute to the negative results that were obtained in studies in which the exercise intervention was initiated 5 days after DA lesion (O'Dell et al., 2007; Petzinger et al., 2007; Hood et al., 2016). As indicated by Table 1, studies reporting a neuroprotective on DA neurons commonly administered exercise before and/or immediately after DA lesion surgery, whereas studies reporting a lack of neuroprotective effect on DA neurons commonly administered exercise after a long delay.

#### Exercise intensity

Low intensity exercise may not trigger the production of enough neuroprotective and neurorestorative molecules. This possibility is clearly indicated by the results of Gerecke et al. (2010) on the impact of exercise dose on the neuroprotective effect on DA neurons. It is possible that high intensity exercise may trigger the production of large amounts of neuroprotective molecules to exert neuroprotective effects.

#### Animal age

Neuroprotection and repair are likely more difficult to obtain in older animals than in young animals (Fox et al., 2001). Neurodegeneration is strongly age-dependent, and DA neurons and their axon terminals in the striatum in old animals are more vulnerable to toxins such as MPTP and 6-OHD (Finnegan et al., 1995). However, both the positive and negative studies reviewed here used young adult (3–4 months of age) mice or rats. Thus, animal age is apparently not a factor contributing to the discrepancy.

## Exercise effects on MSN anatomy, intrinsic physiology and cortical synaptic inputs in animal PD models

### Baseline properties of MSNs

MSNs are a unique class of neurons in the brain. Anatomically, they have well-developed, rich dendritic spines (Kemp and Powell, 1971; Preston et al., 1980; Wilson and Groves, 1980; Bishop et al., 1981; Chang et al., 1981; McNeill et al., 1988; Kawaguchi et al., 1989, 1990; Kincaid et al., 1998; Fujiyama et al., 2011). These dendritic spines are the locations for MSNs to receive and process synaptic inputs, especially glutamatergic inputs from the cerebral cortex and the thalamus (Gerfen and Bolam, 2017). Physiologically, due to tonically active outward K currents such as the inward rectifier, MSNs have a very hyperpolarized resting membrane potential and do not fire spikes unless receiving strong or synchronized excitatory synaptic inputs from the cortex and the thalamus (Wilson and Kawaguchi, 1996; Tseng et al., 2001; Kasanetz et al., 2006; Mahon et al., 2006; Kita and Kita, 2011). The main excitatory synaptic drives to striatal MSNs receive glutamatergic synaptic inputs from the cerebral cortex and thalamus (Deng et al., 2015). MSNs rely on the cortical and thalamic glutamatergic inputs (EPSCs) to trigger spike output (Doig et al., 2010; Huerta-Ocampo et al., 2014; Smith et al., 2014). High levels of D1Rs are expressed near the glutamatergic synapses in the dendrites/spines, indicating that DA/D1 agonism may affect both cortical and thalamic inputs (Moss and Bolam, 2008; Gerfen and Bolam, 2017). D1R agonism may enhance NMDA receptor (NMDA-R)- and AMPA-R-mediated currents in D1-MSNs (André et al., 2010), and D2Rs commonly inhibit NMDA-Rs and AMPA-Rs (Tritsch and Sabatini, 2012).

MSN activity is critical to the motor function in animals including humans. Specifically, activation of the D1-MSNs in the direct pathway facilitates movements, whereas activation of D2-MSNs inhibits movements; coordinated activation of D1-MSNs and D2-MSNs confers the animal with normal motor control function (Bateup et al., 2010; Kravitz et al., 2010; Cui et al., 2013; Friend and Kravitz, 2014; Jin et al., 2014; Tecuapetla et al., 2016). Under parkinsonian condition, D2R-mediated DA inhibition of D2-MSNs is lost such that D2-MSNs become more excitable than under normal condition, thus increasing the spiking activity (Singh et al., 2016; Shi et al., 2017). Thus, MSN activity may be a key target for exercise to affect.

### Exercise effects on MSN spine loss in animal PD models

Studies in human PD brains have indicated a dendritic atrophy and a dendritic spine loss in MSNs (McNeill et al., 1988; Stephens et al., 2005; Zaja-Milatovic et al., 2005). A similar dendritic atrophy and dendritic spine loss have been observed in MPTP monkey PD models (Villalba and Smith, 2011; Villalba et al., 2015) and rodent PD models (Ingham et al., 1998; Day et al., 2006). Enlargment of synapses (number of perforated synapses) was reported to be increased in human PD patients (Muriel et al., 2001), monkey PD model (Villalba and Smith, 2011; Villalba et al., 2015), and rat PD model (Ingham et al., 1998). Given the critical role of striatal MSNs in motor control and other brain functions, an important question is: Does exercise affect MSN structure (dendrites and spines) and function? There is currently no human data on this question and only a few studies in experimental animals. Here we will summarize and discuss the data from these studies.

**Figure 4:**
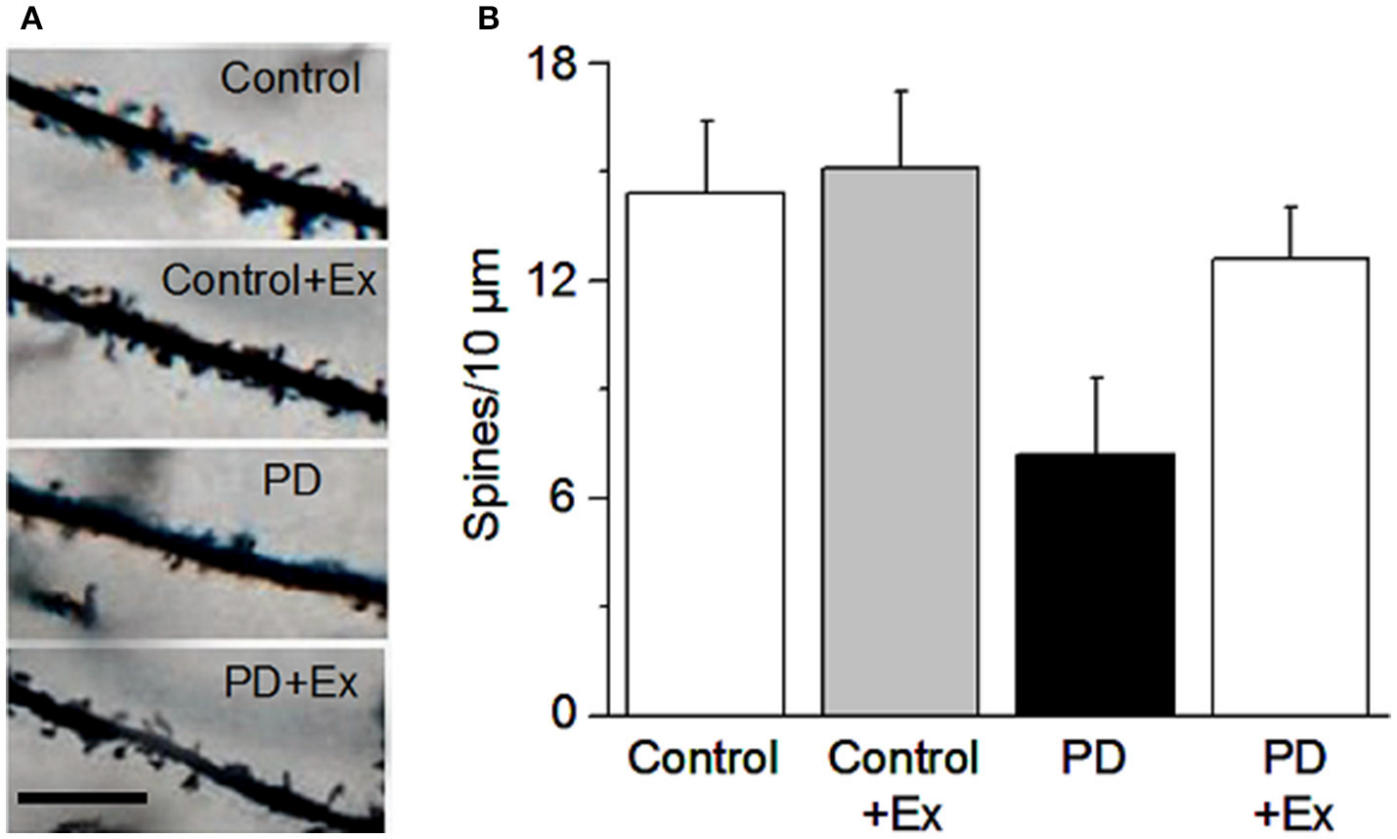
Exercise protects MSN dendritic spines. **(A)** Example dendritic spines under the four conditions. Scale bar, 5 μm. **(B)** Quantification of dendritic spines under the four conditions. Modified from Chen et al. (2015a) with permission.

In mice assessed using the classic Golgi staining method supplemented by intracellular staining, Toy et al. (2014) reported that MPTP DA lesion (90% DA loss) caused a ~20% loss of dendritic spines in both D1- and D2-MSNs; a 6-week intensive treadmill exercise regimen, initiated even 5 days after MPTP lesion, reversed the dendritic spine loss in both D1- and D2-MSNs in the dorsolateral striatum (potentially by forming new spines), enhanced dendritic arborization, and increased the expression of synaptic proteins PSD-95 and synaptophysin, accompanied by a normalization of MPTP lesion-induced motor deficits; however, the striatal tissue DA content was not affected by exercise; instead, the same lab reported previously that exercise decreased DA uptake, thus increasing DA availability (Petzinger et al., 2007). Toy et al. (2014) also reported that a minimum of 5 weeks of the exercise training was needed to reverse the motor deficits with 4 weeks being insufficient. Furthermore, that study also reported that exercise increased MSN dendritic spine density in normal mice, indicating that exercise can promote new spine formation (Toy et al., 2014). Since exercise was initiated 5 days after the 1-day subacute MPTP lesion, i.e., when the toxin-induced lesion was complete, exercise's reverse of dendritic spine loss can be considered a form of neurorestoration or repair. At the Beijing Normal University Exercise Physiology Laboratory, we have also observed that in 6-OHDA-lesioned rats, a 4-week treadmill exercise regimen initiated 1 day after the 6-OHDA lesion surgery partially reversed MSN dendritic spine loss (Chen et al., 2015a) (Figure 4).

In addition to the reported dendritic spine loss in MSN in PD patients and both D1- and D2-MSNs in monkey MPTP PD model, combined immunohistochemical and ultrastructural studies have indicated that corticostriatal and thalamostriatal axospinous synapses on MSN dendritic spines increase their volume and increase PSD size and PSD perforation (more perforated synapses), larger presynaptic glutamatergic axon terminals, providing an anatomical foundation for increased glutamatergic synaptic transmission at these remaining synapses (Villalba and Smith, 2011; Villalba et al., 2015), compensating for the loss of MSN dendritic spines. But this issue is not settled because some studies reported that 6-OHDA lesion only caused spine loss in D2-MSNs but not in D1-MSNs in mice (Day et al., 2006). In MPTP lesioned monkeys, it has been reported that dendritic spine loss are in both D1- and D2-MSNs (Villalba and Smith, 2011; Villalba et al., 2015); yet another study reported that MPTP lesion in monkeys reduced dendritic spines in D2-MSNs and increased dendritic spines in D1-MSNs (Scholz et al., 2008). In PD patient postmortem brains, Anglade et al. (1996) reported that “the size and density of dendritic spines and the size of postsynaptic density perforations were unchanged” in MSNs in the caudate nucleus. Also in PD brains, Muriel et al. (2001) reported a 50% increase in the number of perforated synapses on D1-MSN dendritic spines while seeing no change in the number of perforated synapses on non-D1R (i.e., D2-MSN) spines in striatal neurons. The reasons for these discrepancies are not known. Future studies are needed to establish the anatomical abnormalities in MSNs in PD brains and animal PD models.

At the Exercise Physiology Laboratory at Beijing Normal University, we have also investigated the potential effects of exercise on the structure and function of MSNs in the classic unilateral medial forebrain bundle 6-OHDA injection lesion rat PD model (Ungerstedt, 1971a,b). Twenty-four hours after the lesion surgery, rats in exercise group receive a 4-week exercise intervention, using a motorized treadmill at a speed of 11 m/min (30 min/day, 5 days/week), a common exercise regimen in the literature (e.g., Tajiri et al., 2010). The broad spectrum DA agonist apomorphine was subcutaneously injected to the rats on 7, 14, and 28 days to induce contralateral rotations as an indirect readout of DA lesion in the striatum. After the final behavioral tests, the rats were euthanized and the brains were harvested for anatomical and biochemical examination. Our behavioral tests showed that compared with PD rats, PD rats in the exercise group had fewer apomorphine-induced rotations, indicating lower DA sensitization and more residual DA innervation (Chen et al., 2015a). Additionally, our Golgi staining data indicate that 6-OHDA lesion induced a substantial decrease in the total spine density in the 6-OHDA-lesioned rat striatum (Chen et al., 2015a) (Figure 4); ultrastructural studies using electron microscopy indicated an increased perforated synapses in the these 6-OHDA-lesioned rats (Chen et al., 2015b). Although we did not identify D1- and D2-MSNs, the general decline in spines in our 6-OHDA rat model is consistent with a global spine loss situation reported for MPTP-lesioned monkeys (Villalba and Smith, 2011; Villalba et al., 2015). Further, our Western blotting experiments indicated that 6-OHDA lesion decreased the expression of glutamate NMDAR1 and GluR2 in the striatum. Equally important, our exercise intervention regimen partially reversed the MSN spine loss accompanied with motor behavior improvements (Chen et al., 2015a) (Figure 4).

The potential molecular mechanisms underlying exercise-induced apparent neuroprotection of the MSN spines are discussed below in section Molecular Mechanisms Underlying Exercise-Induced Neuroprotection of the Da Neurons and Corticostriatal Circuit.

### Exercise restores corticostriatal glutamatergic neurotransmission

Since cortical glutamatergic inputs, together with the thalamic glutamatergic inputs, are the main force that drives MSN spiking activity (Kita and Kita, 2011) and may contribute to exercise-induced motoric benefits in PD, we have studied the effects of exercise intervention on the corticostriatal glutamatergic neurotransmission in the unilateral 6-OHDA rat model of PD, using immunohistochemical, biochemical, and ultrastructural techniques (Chen et al., 2015a,b). We found that a 4-week exercise intervention normalized both the increased glutamate level and the decreased NMDA receptor subunit 1 in the dorsal striatum in PD rats, determined by HPLC and biochemical methods, respectively (Chen et al., 2015b). Our electron microscopic ultrastructral studies showed that exercise intervention normalized the DA denervation-induced increase in perforated asymmetric, potentially glutamatergic synapses (Chen et al., 2015b). These results suggest that exercise intervention may normalize DA denervation-induced structural and functional abnormalities at the corticostriatal and/or thalamostriatal synapses, providing a neurobiological basis for exercise intervention to normalize the abnormalities at the corticostriatal synapses, although thalamostriatal synapses may also be normalized; our ongoing research is investigating the potential effects of exercise on the corticostriatal and thalamostriatal synapses in the direct and indirect pathway MSNs.

Additionally, our studies indicate that DA denervation decreased the expression of GluR2 in the striatum in 6-OHDA rat PD model, and our treadmill exercise intervention regimen described above normalized this decrease in GluR2 expression (Chen et al., 2015a), largely consistent with the results of Garcia et al. (2017). GluR2 subunit-lacking AMPA-type glutamate receptors can form Ca-permeable glutamatergic receptor ion channels, conduct inwardly rectifying EPSCs, and Ca may trigger multiple molecular and cellular mechanisms in the MSNs (Cull-Candy et al., 2006; Liu and Savtchouk, 2012; Lalanne et al., 2016; Whitehead et al., 2017). Thus, exercise-induced normalization of GluR2 expression can contribute to the ultrastructural normalization of MSNs.

Similar to our results in 6-OHDA rat model, two previous studies have reported that in a subacute MPTP PD mouse model, treadmill exercise, initiated 5 days after MPTP lesion, increased the expression of GluR2 subunit in the striatum; this exercise also reduced the size of corticostriatal EPSCs (glutamatergic input), particularly in D2-MSNs; and these exercise-induced cellular changes were accompanied by behavioral improvements (VanLeeuwen et al., 2010; Kintz et al., 2013).

In aggregate, these findings suggest that exercise may modify and normalize the corticostriatal excitatory synaptic transmission and reduce potential glutamatergic excitatoxicity in the striatum, therefore contributing to the normalization of MSN structure and function such as the dendritic spines and synaptic inputs and processing at dendritic spines, eventually leading to circuittry and behavioral restoration. Functionally, the restored/increased dendrite spines can more effectively receive and process synaptic inputs from the cortical and thalamic motor command centers, contributing to the recovery/normalization of motor functions in these PD animals.

How exercise restores and repairs dendritic spines under normal and PD conditions is not established and will be discussed in section Molecular Mechanisms Underlying Exercise-Induced Neuroprotection of the Da Neurons and Corticostriatal Circuit.

### Exercise effects on MSN physiology in PD

MSN activity and physiology are critical to motor and other important brain functions (see section Motor Function of the Striatum and the Nigrostriatal DA System). Thus, exercise-induced motoric benefits in PD patients and animal PD models may derive at least partially from exercise's potential effects on MSN activity. There is currently no human study on this topic. To our knowledge, there is only one published study investigating the potential effects of exercise on MSN spiking activity in unilateral MFB 6-OHDA PD rats, performed in our lab (Shi et al., 2017). In our study, the 4-week exercise intervention program was initiated 24 h after the 6-OHDA lesion surgery, treadmill speed was 11 m/min, the rat was exercised 30 min/day, 5 days/week. Under these conditions, we found that in 6-OHDA PD group, MSNs had an increased average firing rate (about 3 Hz) compared with normal rats (about 0.5 Hz). This is generally consistent with the literature on DA depletion on MSN firing (Kish et al., 1999; Chen et al., 2001; Singh et al., 2016). Further, in the 6-OHDA+ exercise group, MSN firing was partially normalized (about 2 Hz), accompanied by motor function improvement. Together, these results suggest that exercise may decrease striatal neuron excitability and partially normalize the abnormal neuronal spike firing in parkinsonian striatum, potentially contributing to exercise's motor-improving effects in PD. Although how this partial normalization is achieved remains to be determined, multiple mechanisms may be involved, such as attenuated DA loss and consequent reduction in striatal neuron intrinsic excitability and modification of intrastriatal circuitry (Wei et al., 2017); exercise may also affect the cortical glutamatergic inputs, thus reducing the abnormality in striatal neuron firing (VanLeeuwen et al., 2010; Kintz et al., 2013).

## Molecular mechanisms underlying exercise-induced neuroprotection of the DA neurons and corticostriatal circuit

In the preceding sections, we summarized and discussed the reported physical exercise-induced attenuation of the loss of nigral DA neurons and the loss of striatal DA axons and DA tissue level in the striatum in 6-OHDA and MPTP animal PD models. Now we discuss the possible underlying molecular mechanisms. The attenuation of DA denervation may be realized via two different though related mechanisms: neuroprotection, neurorestoration or both. In the neuroprotection scenario, the neurons are protected from toxin insults before the injury is done. In the neurorestoration scenario, the neurons are first injured and then repaired. These two mechanisms probably work together in animals, for example, IP injection of MPTP in mice often injures DA neurons and DA axon terminals in the striatum; these sick DA neurons and axons often recover after the cessation of MPTP treatment, especially in young mice.

The molecular mechanisms underlying the exercise-induced protection and restoration of the nigrostriatal neurons and corticostriatal circuit are not established, but evidence indicates that neurotrophic factors are potentially critical mediators of these beneficial effects (da Silva et al., 2016); other molecules and mechanisms also contribute, such as reducing oxidative stress, increasing energy production and mitochondrial function, and increased blood flow via vasodilatation and angionesis (Petzinger et al., 2013; Nishijima et al., 2016). Studies have reported that exercise improved mitochondrial function, reduced α-synuclein expression and reduced the production of pro-inflammatory factors in MPTP PD model, leading to attenuated DA denervation and motor function loss (Sung et al., 2012; Subramaniam and Chesselet, 2013; Goes et al., 2014; Kelly et al., 2014; Spielman et al., 2016; Jang et al., 2017; Koo et al., 2017a,b). Here, we focus on neurotrophic factors (NTFs) because exercise increases NTFs in normal animals and humans and NTFs are neuroprotective and neurorestorative (Cotman et al., 2007; Voss et al., 2013; Arnold and Salvatore, 2016).

### Neurotrophic factors (NTFs) in the nigrostriatal DA system

Stimulation of nerve cell growth or neurotrophic effect exerted by biological agents (now referred to as neurotrophic factors) was first observed in cultured peripheral nerve cells in the early 1950s (Levi-Montalcini and Hamburger, 1951; Levi-Montalcini and Cohen, 1956). It is now established that NTFs are secreted proteins that are critical to the growth, development, maturation, maintenance, plasticity, and repair and regrowth of the peripheral and central nervous system neurons (Barde et al., 1982; Leibrock et al., 1989; Hou et al., 1996; Levi-Montalcini et al., 1996; Airaksinen and Saarma, 2002; Bespalov and Saarma, 2007; Park and Poo, 2013; Kramer and Liss, 2015; Ibáñez and Andressoo, 2017; Sasi et al., 2017). Currently, more than 20 NTFs have been identified, and these NTFs are divided into four groups according to their molecular structure and signaling mechanisms: the neurotrophine group including the extensively studied NGF and BDNF, the GDNF family ligands with GDNF being the extensively studied member, the newly found NTF group including cerebral dopamine neurotrophic factor (CDNF) and mesencephalic astrocyte-derived neurotrophic factor (MANF), and the neurokine family (Lindholm et al., 2007; Aron and Klein, 2011; Allen et al., 2013; Ibáñez and Andressoo, 2017; Lindahl et al., 2017).

#### NTFs and DA neurons

In 1993, a glia cell-secreted nerve growth-stimulating factor (termed glial cell line-derived neurotrophic factor or GDNF) was purified, sequenced, and cloned (Lin et al., 1993). Further, that study reported that GDNF strongly promotes the survival of rat midbrain DA neurons in tissue culture, raising the hope that GDNF may affect the survival and degeneration of DA neurons in health and PD in humans, and hence GDNF can be used to stop DA neuron degeneration in PD. Thus, a large number of *in vitro* and *in vivo* studies were quickly performed, as reflected in the publication of four original research papers on GDNF saving DA neurons and motor neurons in the January 26th, 1995, issue of Nature (DA neurons: Beck et al., 1995; Tomac et al., 1995; motor neurons: Oppenheim et al., 1995; Yan et al., 1995). Specifically, it was reported that GDNF injected into the nigral area or striatum reduced the MPTP- or axotomy-induced DA cell loss in the SNc and DA denervation in the striatum (Beck et al., 1995; Tomac et al., 1995). Further, upon inhibition of BDNF and GDNF expression, the DA axon sprouting in the knife-lesioned striatum was reduced in mice (Batchelor et al., 2000). It has also been reported that conditional GDNF deletion performed in adult mice led to DA neuron loss in SNc and VTA and DA denervation in the striatum and a loss of motor function, indicating that the survival of DA neurons is strongly dependent on GDNF (Pascual et al., 2008); a more recent study has reported contradicting findings (Kopra et al., 2015), although Pascual and López-Barneo (2015) argued that negative results are due to technical problems such as insufficient decrease of GDNF in the new study. Additional follow-up studies investigated and established the neuroprotective effects of GDNF protein or GDNF gene vectors on DA neurons in rodent and monkey PD models (Gash et al., 1995, 1996; Choi-Lundberg et al., 1997; Kordower et al., 2000; Nakajima et al., 2001; Grondin et al., 2002; Maswood et al., 2002; Ai et al., 2003; Kirik et al., 2004; Rangasamy et al., 2010). Taken together, these studies provide strong data supporting GDNF's neurotrophic and neuroprotective effects on DA neurons.

Studies indicate that BDNF also exerts trophic effects on the development, maturation, repair and plasticityof DA neurons (Hyman et al., 1991, 1994; Levivier et al., 1995; Spenger et al., 1995; Ostergaard et al., 1996; Benisty et al., 1998; Numan and Seroogy, 1999; Baker et al., 2005; Baquet et al., 2005; Sun et al., 2005; Baydyuk et al., 2011a,b). Genetic inhibition of BDNF expression led to loss of nigral DA neurons in rats (Porritt et al., 2005). However, evidence indicates that BDNF's trophic effect on nigral DA neurons is 5–10 weaker than that of GDNF (Lu and Hagg, 1997; Sun et al., 2005). BDNF expression in the brain is very low compared with that of NGF (Hofer et al., 1990). Thus, translational research on NTFs' neuroprotective and neurorestorative effects on DA neurons in PD monkey models and clinical trials have been focused on GDNF (Gash et al., 1995, 1996; Kordower et al., 2000; Kozlowski et al., 2000; Connor et al., 2001; Grondin et al., 2002; Maswood et al., 2002; Ai et al., 2003; Gill et al., 2003; Cohen et al., 2011; Kordower and Bjorklund, 2013; Olanow et al., 2015; Bartus and Johnson, 2017a,b; Kirik et al., 2017).

#### NTFs and striatal MSNs

A large number of studies have established that NTF signaling regulates somatic, dendritic and axonal local protein synthesis and gene transcripts and hence support the development, maturation, maintenance, reorganization and regeneration/repair of neuronal somata, axons, dendrites, and dendritic spines and synapses in the striatum and other brain areas (Xu et al., 2000; Horch and Katz, 2002; Gorski et al., 2003; Baquet et al., 2004; Vigers et al., 2012; Lu et al., 2013; Orefice et al., 2013, 2016; Bramham and Panja, 2014; Leal et al., 2014; Zagrebelsky and Korte, 2014). In the striatum, BDNF has been shown to be critical to the survival and maintenance of MSNs (Baydyuk and Xu, 2014).

Evidence from cortical ablation experiments and molecular genetic manipulation experiments and the fact that the cerebral cortex expresses a high level of BDNF mRNA whereas the striatum expresses a very low level of BDNF mRNA, indicating that the majority of striatal BDNF may be produced in cortical neurons and a small portion of striatal BDNF may be produced in substantia nigral neurons, and BDNF is anterogradely transported to the striatum by the corticostriatal axons (hence corticostriatal synapses) (Hofer et al., 1990; Altar et al., 1997; Conner et al., 1997; Baquet et al., 2004; Zuccato and Cattaneo, 2007; Li Y. et al., 2012). Further, conditional tissue-specific genetic deletion of bdnf gene in the cerebral cortex and the substantia nigra completely depleted BDNF protein in the striatum, confirming that striatal BDNF protein is transported to the striatum from the cerebral cortex and substantia nigra (Li Y. et al., 2012); equally important, this BDNF depletion led to smaller MSN soma size, atrophy, and loss of dendrites and dendritic spines, a lower expression of DARPP-32 (a key mediator of DA signaling) and severe motor deficits (Baquet et al., 2004; Li Y. et al., 2012). Similarly, genetic inactivation of BDNF receptor TrkB selectively in the striatal MSNs led to smaller MSN size, dendritic spine loss and a greatly reduced DARPP-32 level and also a lower level of TH expression in DA axons (Li Y. et al., 2012). Global deletion of BDNF or TrkB led to neuronal (somata, dendrite, spine) atrophy in multiple brain areas and the striatal MSNs are particularly sensitive, leading to a substantial MSN dendrite and spine atrophy (Rauskolb et al., 2010; Li Y. et al., 2012).

Taken together, the NTF mechanisms discussed above provide opportunities for exercise to affect MSN dendritic spines and corticostriatal synapses and also nigral DA neurons and their axon terminals via exercise-stimulated NTF production.

### Neurotrophic factors in PD

#### NTF deficiency in PD brains

There are only a few limited studies on this important question. These studies indicate a deficiency in NTFs in PD brains. Quantitative histochemical studies have reported that BDNF gene expression and protein are reduced in the substantia nigra in postmortem PD brains (Mogi et al., 1999; Parain et al., 1999; Howells et al., 2000). The GDNF level may also be reduced in the PD brain (Chauhan et al., 2001), although the numbers of the brains included in these studies were relatively small. Future studies are needed that will include larger samples from different stages (to compare early and late stages; early stage maybe particularly informative) to replicate, solidify and expand these results.

### NTF neuroprotective effects in animal PD models

Since NTFs are intrinsically beneficial to DA neurons and MSNs and NTFs may be deficient in PD brains, an obvious idea is that increasing the production of neurotrophic factors may be neuroprotective and neurorestorative for PD brains. Indeed, since the early 1990s, it has been the focus of a large basic and clinical research effort to directly deliver NTF proteins and more recently implanting genetically engineered NTF-producing cells or gene vectors to locally produce NTFs and protect DA neurons in rodent and non-human primate PD models and patients (Olson et al., 1991; Lin et al., 1993; Kordower et al., 2000; Kozlowski et al., 2000; Gill et al., 2003; Cohen et al., 2011; Nagahara and Tuszynski, 2011; Kordower and Bjorklund, 2013; Olanow et al., 2015; Li et al., 2016; Bartus and Johnson, 2017a,b; Björklund and Lindvall, 2017).

In addition to the classical neurotrophic factors, the newly discovered conserved cerebral dopamine neurotrophic factor (CDNF) and mesencephalic astrocyte-derived neurotrophic factor (MANF) have also been reported to have trophic effects on nigral DA neurons in normal animals and neuroprotective effects on these neurons in animal PD models (Lindholm et al., 2007; Garea-Rodríguez et al., 2016; Lindahl et al., 2017).

#### NTF neuroprotective effects in clinical trials in PD patients

The positive neuroprotective and neurorestoration effects of NTFs in animal PD models prompted clinical trials of NTFs in PD patients in the past three decades. Open label NTF trials produced positive results (e.g., Olson et al., 1991; Gill et al., 2003; Slevin et al., 2005). However, more rigorous double blind clinical trials failed to prove any NTF benefit in PD (Kordower and Bjorklund, 2013; Olanow et al., 2015; Bartus and Johnson, 2017a; Hegarty et al., 2017). Two reasons may have contributed to this failure. First, the exogenous NTF (mostly GDNF)–protein or gene-is supplied by a point source and hence does not provide enough NTF to the target tissue (the striatum and SNc) (Aebischer and Ridet, 2001; Gash et al., 2005; Salvatore et al., 2006). Further, NTFs can not cross the blood brain barrier, diffuses poorly and are easily degraded (Aron and Klein, 2011). Thus, the failure of these clinical trials may be a delivery problem, not because NTFs are ineffective. Second, the procedure is an invasive intracranial surgery with considerable risks and not acceptable to early-mid stage PD patients who still have considerable residual DA neurons and their striatal projection axons that can be protected by NTFs; in late stage PD patients who may accept the invasive procedure, the nigrostriatal DA system is almost completely destroyed and nothing can be done.

Therefore, an early-start, low-risk neuroprotection program is needed to protect the vulnerable DA neurons and other neuron types, slow the rate of their degeneration and hence delay the appearance of PD symptoms. Exercise is non-invasive, and risk/side effect-free stimulator of NTF production with benefits to many organ-systems. Thus, exercise may be such an early-start neuroprotective and neurorestorative treatment for PD.

### Exercise stimulates NTF production in PD

Besides supplying exogenous NTFs via an invasive surgery-infusion means, other methods that stimulate the production of endogenous NTFs can also be neuroprotective while avoiding the risks associated with intracranial infusion surgery. Research since the 1990's has indicated that physical activity or exercise can increase NTF production in normal animals and also in humans, although human studies are few and only serum NTFs were tested due to the difficulties in obtaining human tissue samples (Neeper et al., 1995, 1996; van Praag et al., 1999, 2005; Cotman and Berchtold, 2002; Cotman et al., 2007; Pereira et al., 2007; Voss et al., 2013; Coelho et al., 2014; Arnold and Salvatore, 2016; Marston et al., 2017).

### Exercise stimulates NTF production in PD patients

There is no direct data on the possibility of exercise stimulating NTF production in the nigrostriatal DA system because it is currently impossible to measure NTFs in brain tissues in PD patients. There is also currently no data from postmortem brain tissues because of the difficulties in obtaining appropriate human brain tissue samples. However, studies have investigated exercise's effects on NTFs in peripheral tissues, particularly in blood. It has been reported that intensive exercise increased blood BDNF levels and increased TrkB activity in blood cells, although data on brain tissues are lacking due to the obvious difficulties in obtaining brain tissue samples (Frazzitta et al., 2014; Angelucci et al., 2016; Fontanesi et al., 2016). Independently, it has been reported that the basal serum BDNF level was lower in PD patients than in normal control (Scalzo et al., 2010), and exercise increases the production of BDNF and other NTFs in PD patients (Zoladz et al., 2014; Marusiak et al., 2015). If we extrapolate these peripheral findings, then exercise may stimulate the endogenous production of NTFs in the brain that in turn exert neuroprotective and neurorestorative effects on DA neurons, modify/slow the disease progression. Certainly, future studies need to experimentally verify this extrapolation.

### Experimental data from PD animal models

To circumvent the difficulties in studying PD patients, researchers have used animal PD models to investigate how exercise affects NTF production in the brain that may in turn exert neuroprotective and neurorestorative effects on DA neurons and MSNs associated with behavioral benefits in PD animals (da Silva et al., 2016). For example, in a chronic, low-moderate dose MPTP mouse model with moderate nigral DA neuron loss and striatal DA loss (Lau et al., 2011), treadmill exercise before, during and after MPTP treatment partially prevented the loss of nigral DA neurons and striatal TH and DA, compared to similarly lesioned sedentary mice, accompanied by a prevention of motor function deficits. In this mouse model, exercise also increased the tissue level of BDNF in the nigral area and the GDNF level in both the nigral area and striatum; exercise also partially normalized the mitochondrial function as indicated by increased ATP level in the striatal tissue in exercised MPTP-lesioned mice than in sedentary MPTP-lesioned mice (Lau et al., 2011). Tajiri et al. (2010) reported that exercise substantially increased striatal tissue BDNF and GDNF (by ~100%) in normal rats, increased striatal tissue BDNF and GDNF level by 50% in PD rats compared with non-exercised PD rats, measured by western blot. In both mouse and rat intrastriatal 6-OHDA lesion PD models a pre-lesion, 60-day treadmill exercise increased and hence partially normalized the striatal tissue levels of proBDNF, BDNF and its receptor TrkB, increasing striatal tissue BDNF level by 33% in PD animals compared with non-exercised PD animals, measured by western blot, accompanied by parallel behavioral improvements (Tuon et al., 2012, 2014). This pre-lesion physical exercise also partially prevented or normalized the intrastriatal 6-OHDA-induced reduction in TH (i.e., DA axon loss) in the striatum in rats (Tuon et al., 2012). Further, exercise's apparent protective effect on DA neurons was reduced when BDNF receptors were blocked (Real et al., 2013). Additionally, it has been reported that a reduced BDNF expression in haploinsufficient BDNF^+/−^ mice eliminated exercise-induced protection of the nigrostriatal DA neurons against MPTP neurotoxicity (Gerecke et al., 2012). Besides BDNF and GDNF, exercise may also trigger the production of other trophic factors that may also be involved in exercise-induced neuroprotection in PD animals (da Silva et al., 2016). Together, these studies indicate that exercise-induced protection of DA neurons is dependent on NTF production.

Besides protecting DA neurons from toxin and other insults before the damages are done or completed, another mechanism underlying the exercise's benefits in PD is for exercise to help residual DA axons in the striatum to repair and/or regenerate-i.e., neurorestoration, potentially via the same NTF mechanisms discussed above and other mechanisms not covered in this review such as increasing blood flow and mitochondrial function and decreasing oxidative stress and inflammatory factors. Use/exercise-induced facilitation of neurorestoration is well-documented in the neurorehabilitation literature (e.g., Jones and Schallert, 1994; Schallert et al., 1997, 2000; Takamatsu et al., 2010; Korol et al., 2013; Tamakoshi et al., 2014). Further, neuroprotection and neurorestoration are likely intertwined especially when examined 1–2 months after the toxin administration, both processes lead to attenuated DA axon loss in the striatum and/or attenuated DA neuron loss in the nigral areas. The repairing/regeneration/neurorestoration idea is particularly attractive because the striatal DA axons are known to have to capacity to regenerate in 6-OHDA and MPTP rodent and non-human primate PD models. For example, it has been reported that when the 6-OHDA lesion is ~65% DA denervation in the striatum, DA axons may sprout and regenerate in rodents and non-human primates, but no sprouting/regeneration was observed when the DA denervation is more complete ≥90% (Liberatore et al., 1999; Bezard et al., 2000; Elsworth et al., 2000; Finkelstein et al., 2000; Stanic et al., 2003a,b; Petzinger et al., 2006; Mounayar et al., 2007; Lee et al., 2008). Song and Haber (2000) also reported that residual DA axons sprout in the striatum in MPTP-lesioned monkeys. These suggest that DA neuron neuroprotection and regeneration are possible before the DA neurons are completely dead/destroyed. Thus, exercise, via NTFs and other molecules, may facilitate the innate capacity of DA neurons and axons to sprout, repair and regenerate, depending on DA lesion protocol, intensity, exercise protocol/timing. When the DA lesion is too severe, it may be impossible to obtain exercise-induced neuroprotection of DA neurons and DA axons in the striatum; when the DA lesion is moderate, exercise-induced DA neuron protection is less difficult to realize.

## Conclusions and future directions

**Figure 5:**
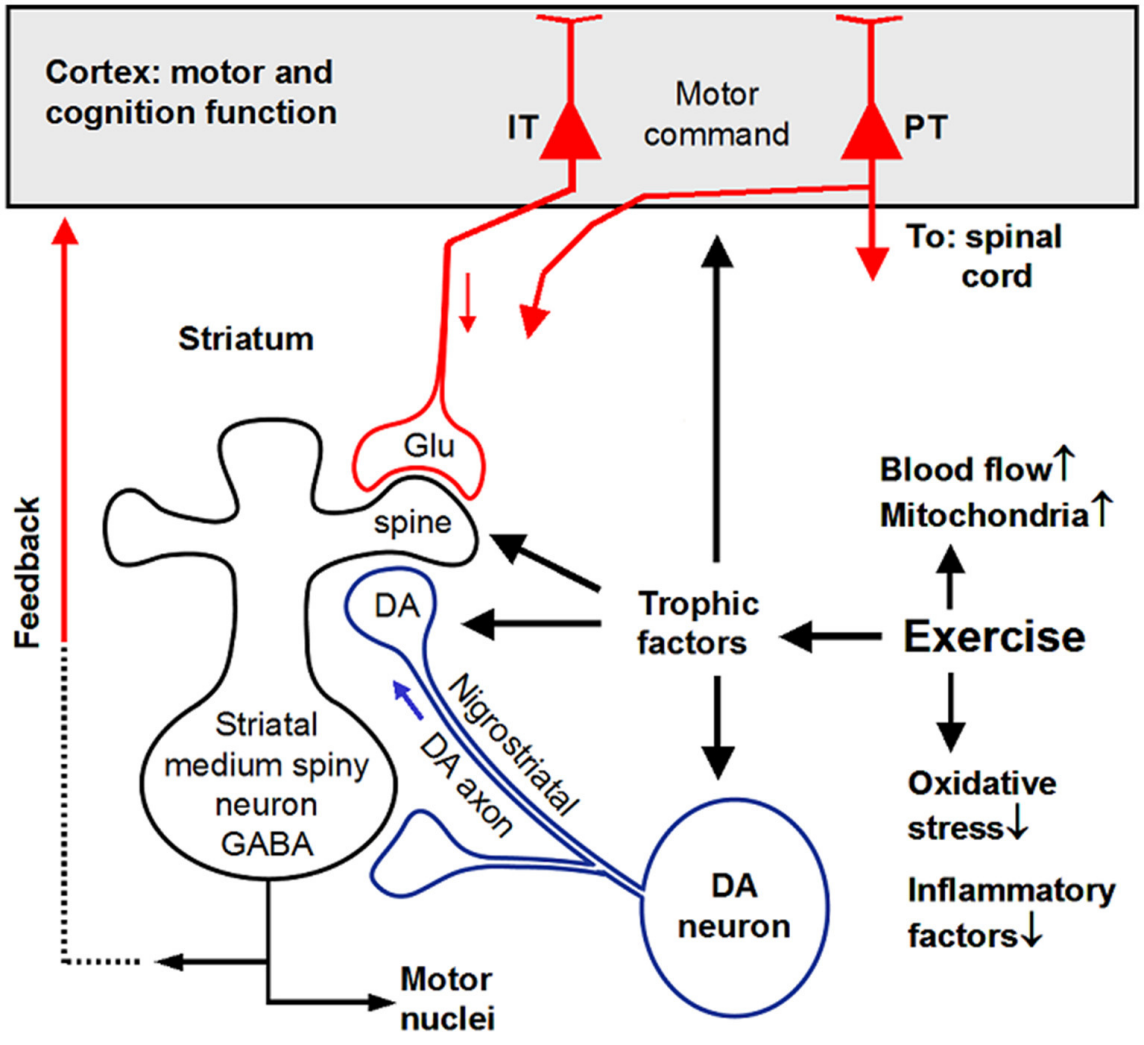
Summary diagram showing that exercise triggers trophic factor production that in turn induce neuroprotection and neurorestoration. IT, intratelencephalically projecting cortical neurons; PT, pyramidal tract projecting cortical neurons. Original artwork of Fu-Ming Zhou.

In conclusion, epidemiological data indicate that exercise may reduce the risk of developing PD and slow PD progression (Figure 5). Clinical evidence indicates that exercise may be a convenient, non-invasive, side effect-free, cost-free treatment that is beneficial to PD patient's motor and cognitive functions. Thus, exercise should be prescribed to PD patients. Experimental data indicate, as illustrated in Figure 5, that exercise may protect and restore the nigrostriatal DA system and the corticostriatal synapse, hence restoring motor and other behavioral functions, potentially by triggering the production of trophic factors and therefore protecting and restoring DA neurons, particularly their massive and distant and hence vulnerable axonal arborization in the striatum. Exercise-stimulated trophic factors may also protect and restore the MSN-based BG-cortical circuits, improving motor, and cognitive functions.

To advance the field of exercise-induced neuroprotection of DA neurons in PD, an important task for basic science research is to establish reliable and standardized protocols to produce exercise-induced protection of DA neurons in rodent and non-human primate PD models, thus resolving the major discrepancy that while a large number of studies saw exercise-induced neuroprotection of DA neurons in animal PD models, several studies did not. We also need to determine the anatomical changes of the DA axon terminals and somata during exercise-induced neuroprotection, elucidating the anatomical substrate for the functional recovery.

For translational and clinical research, we need to determine, in humans, which form of exercise is most effective in reducing PD risk, when exercise should start that will produce protection against PD and DA loss. In parallel experimental studies in animals, we need to determine when exercise should start to produce the maximal DA neuron protection in animal PD models. The dose (exercise intensity)-response (behavioral, anatomical, and neurochemical benefits) relation also needs to be established in both animals and humans. These mundane and incremental studies will build a solid foundation for this clinically important field to move forward.

## Author contributions

LH, WC, XL and DQ: drafting and editing. F-MZ: conceptualization, drafting, and editing.

### Conflict of interest statement

The authors declare that the research was conducted in the absence of any commercial or financial relationships that could be construed as a potential conflict of interest.
